# A new species of *Gamasomorpha* Karsch, 1881 (Araneae, Oonopidae) from Yunnan Province, China

**DOI:** 10.3897/BDJ.14.e183760

**Published:** 2026-02-18

**Authors:** Kuiwen Yang, Yanfeng Tong, Zizhong Yang

**Affiliations:** 1 Basic Medical College, Jinzhou Medical University, Jinzhou 121001, China Basic Medical College, Jinzhou Medical University Jinzhou 121001 China; 2 College of Life Science, Shenyang Normal University, Shenyang 110034, China College of Life Science, Shenyang Normal University Shenyang 110034 China; 3 National-Local Joint Engineering Research Center of Entomoceutics, Dali University, Dali 671000, China National-Local Joint Engineering Research Center of Entomoceutics, Dali University Dali 671000 China

**Keywords:** biodiversity, new taxa, taxonomy, Asia

## Abstract

**Background:**

The oonopid spider genus, *Gamasomorpha* Karsch, 1881, currently comprises eight species known from China, of which one is from Yunnan, namely *G. barbifera* Tong & Li, 2007, recorded from the Ailao Mountain and Diancang Mountain.

**New information:**

A new species, *Gamasomorpha changning* sp. nov. (♂♀), is described, based on specimens collected from Yunnan Province. Morphological descriptions and photomicroscopy images of the new species are provided.

## Introduction

The oonopid spider genus *Gamasomorpha* Karsch, 1881 currently comprises 52 species and has a wide distribution, ranging from South America and Africa to Indonesia, China and Australia (WSC 2025). This genus is characterised by well-sclerotised scuta, a reddish-brown body, spineless legs, a palpal bulb not fused with the cymbium and a long, slender embolus approximately as long as the bulb (Eichenberger et al. 2012). Several species are evidently misplaced in this genus and a comprehensive revision is therefore essential (Bai et al. 2025).

To date, eight species of *Gamasomorpha* have been recorded from China, namely *G. anhuiensis* Song & Xu, 1984 (Anhui), *G. barbifera* Tong & Li, 2007 (Yunnan), *G. cataphracta* Karsch, 1881 (Southern China), *G. comosa* Tong & Li, 2009 (Hainan), *G. linzhiensis* Hu, 2001 (Xizang), *G. nigrilineata* Xu, 1986 (Anhui, Zhejiang), *G. shoushanensis* (Tong & Li, 2014) (Taiwan) and *G. virgulata* Tong & Li, 2009 (Hainan) (Tong and Li 2009, Tong and Li 2014, Bai et al. 2025). Despite these records, the genus remains poorly studied in China and additional species are likely to be discovered through continued fieldwork and taxonomic research.

In this paper, a new species of *Gamasomorpha*, collected from Yunnan Province of China, is described and illustrated.

## Materials and methods

The specimens were examined using a Leica M205C stereomicroscope. Details were studied under an Olympus BX51 compound microscope. Photomicroscope images were made with a Canon EOS 750D zoom digital camera (18 megapixels) mounted on an Olympus BX51 compound microscope. Photos were stacked with Helicon Focus 6.7.1 and processed in Adobe Photoshop CC 2020. Scanning electron microscope images (SEM) were obtained under high vacuum using a Hitachi S-4800 after critical-point drying and gold-palladium coating. All measurements were taken using an Olympus BX51 compound microscope and are in millimetres. Taxonomic descriptions follow Eichenberger et al. (2012) and Tong and Li (2014). The distribution map was generated with ArcGIS v. 10.2 (ESRI Inc.). The type material is deposited in the College of Life Science, Shenyang Normal University (**SYNU**) in Liaoning, China.

The following abbreviations are used in the text: ALE = anterior lateral eyes; boc = booklung covers; ce = conical extension; co = conductor; em = embolus; gap = globular appendix; ma = mesal embolic accessory appendage; na = nail-like process; PLE = posterior lateral eyes; PME = posterior median eyes; psc = paddle-like sclerite; re = receptacle; scr = scutal ridge.

## Taxon treatments

### Gamasomorpha changning sp. nov.

BE827EB8-719A-518E-A281-182D21F7FC46

AEEC7242-6509-4E3C-B629-EBBAD3EA59B4

#### Materials

- **Type status:** Holotype. **Occurrence:** recordedBy: Zizhong Yang & Haibo Pu; individualCount: 1; sex: male; lifeStage: adult; occurrenceID: FAA654C1-B9BC-5D3C-9D1B-E08BB91B23DB; **Taxon:** scientificName: *Gamasomorpha changning*; order: Araneae; family: Oonopidae; genus: Gamasomorpha; scientificNameAuthorship: Yang, Tong & Yang; **Location:** country: China; stateProvince: Yunnan; county: Changning; locality: Mangshui Town, Donghoushan Village, under tree bark; verbatimElevation: 1833 m; verbatimCoordinates: 24°54′20.0″N，99°12′51.9″E; **Event:** eventDate: 2-Oct-2011
- **Type status:** Paratype. **Occurrence:** recordedBy: Zizhong Yang; individualCount: 1; sex: female; lifeStage: adult; occurrenceID: 0C833DF3-1580-538A-A2E7-43E4E12C2B7A; **Taxon:** scientificName: *Gamasomorpha changning*; order: Araneae; family: Oonopidae; genus: Gamasomorpha; scientificNameAuthorship: Yang, Tong & Yang; **Location:** country: China; stateProvince: Yunnan; county: Dali City; locality: Cangshan Mt., Jishejing, pitfall trapping in forest; verbatimElevation: 2700 m; verbatimCoordinates: 25°34'06.493"N, 100°08'23.914"E; **Event:** eventDate: 25-Jul-2009
- **Type status:** Paratype. **Occurrence:** recordedBy: Zizhong Yang; individualCount: 1; sex: female; lifeStage: adult; occurrenceID: F56C6E5C-520A-548A-A13A-D895B8B53BF7; **Taxon:** scientificName: *Gamasomorpha changning*; order: Araneae; family: Oonopidae; genus: Gamasomorpha; scientificNameAuthorship: Yang, Tong & Yang; **Location:** country: China; stateProvince: Yunnan; county: Dali City; locality: Cangshan Mt., Jishejing, pitfall trapping in forest; verbatimElevation: 2700 m; verbatimCoordinates: 25°34'06.493"N, 100°08'23.914"E; **Event:** eventDate: 25-Jul-2009

#### Description

**Figure 1.**
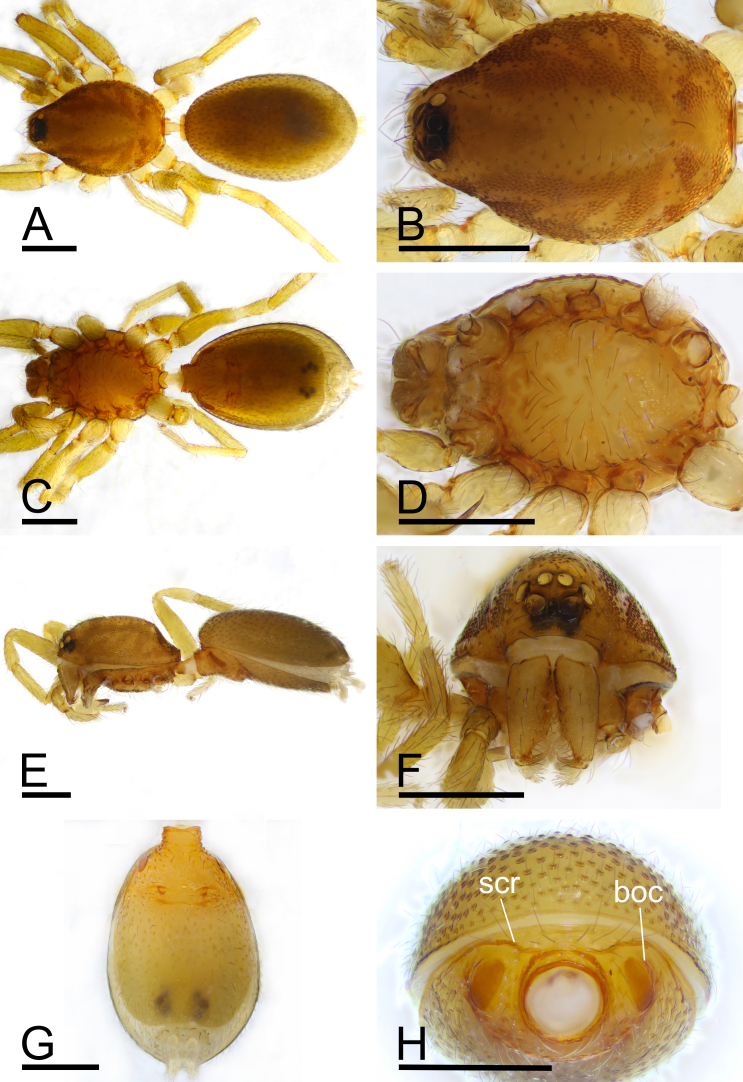
*Gamasomorpha changning* sp. nov., holotype male. **A** habitus, dorsal view; **B** cephalothorax, dorsal view; **C** habitus, ventral view; **D** cephalothorax, ventral view; **E** habitus, lateral view; **F** cephalothorax, anterior view; **G** abdomen ventral view; **H** abdomen, anterior view. Abbreviations: boc = booklung covers; scr = scutal ridge. Scale bars: 0.4 mm (A–H).

**Male**. Total length 2.28; carapace 1.01 long, 0.72 wide; abdomen 1.27 long, 0.79 wide. Habitus as in Fig. 1A, C and E. Body yellowish-brown, legs yellow. Carapace (Fig. 1B and F): elongated oval, pars cephalica slightly elevated in lateral view; dorsal surface smooth, sides strongly granulated. Eyes (Fig. 1B and F): ALE largest, PLE and PME subequal; posterior eye row recurved in dorsal view, procurved in anterior view; PME almost touching, separated from PLE by about PME radius; ALE separated by about their radius; ALE separated from edge of carapace by about their diameter. Sternum (Fig. 1D): longer than wide, with narrow, transverse palpal groove, radial furrows present. Abdomen (Fig. 1G and H): dorsal scutum ovoid, punctate, densely covered with short setae; booklung covers large; pedicel tube short, without dorsolateral extensions; scuto-pedicel region with scutal ridge. Palps (Fig. 3A–C and Fig. 4A–F): pale orange; cymbium not extending beyond distal tip of bulb; bulb distally tapering, ending as round conical extension (ce); embolus (em) long, slender, lamellar, with an opening subapically; embolus positioned adjacent to an embolic accessory appendage (ma) and a lamellar conductor (co).

**Figure 2.**
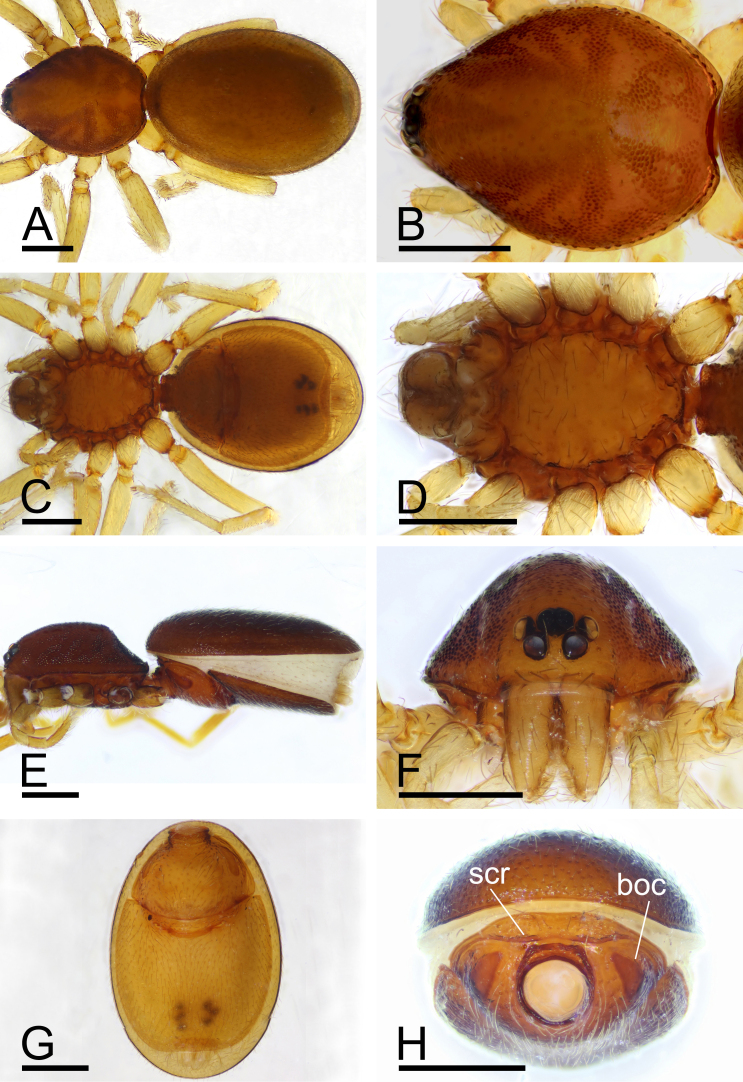
*Gamasomorpha changning* sp. nov., paratype female. **A** habitus, dorsal view; **B** cephalothorax, dorsal view; **C** habitus, ventral view; **D** cephalothorax, ventral view; **E** habitus, lateral view; **F** cephalothorax, anterior view; **G** abdomen, ventral view; **H** abdomen, anterior view. Abbreviations: boc = booklung covers; scr = scutal ridge. Scale bars: 0.4 mm (A–H).

**Figure 3.**
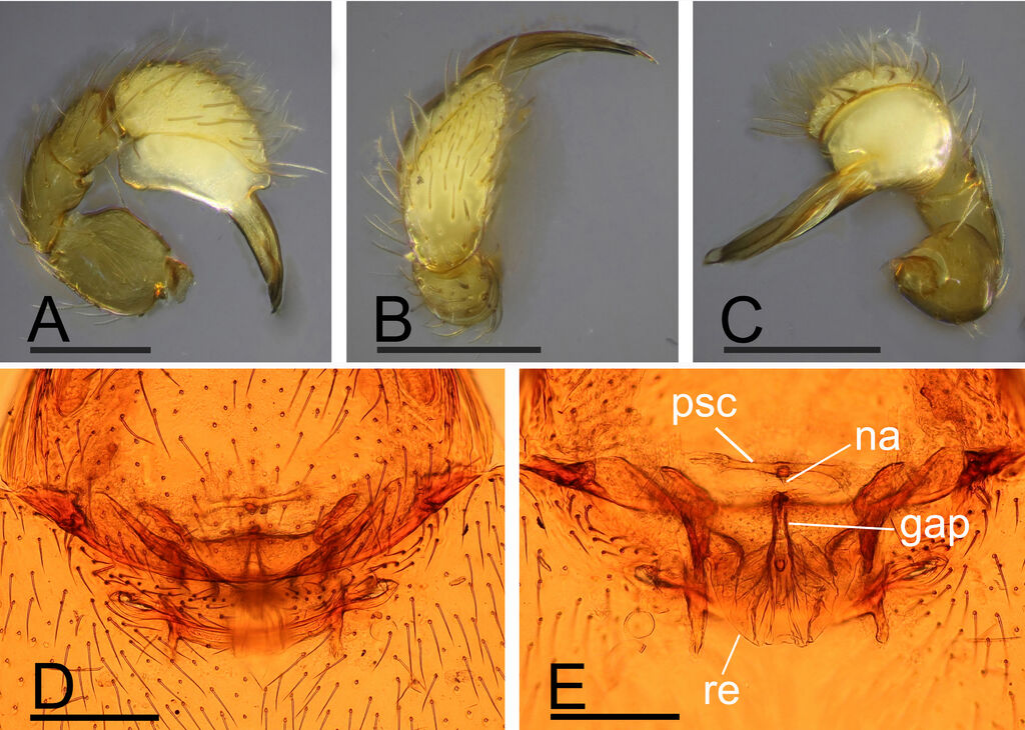
*Gamasomorpha changning* sp. nov. **A** male palp, prolateral view; **B** same, distal view; **C** same, retroventral view; **D** female epigastric region, ventral view; **E** endogyne, dorsal view. Abbreviations: gap = globular appendix; na = nail-like process; psc = paddle-like sclerite; re = receptacle. Scale bars: 0.1 mm (A–C); 0.2 mm (D–E).

**Female**. Total length 2.69; carapace 1.01 long, 0.89 wide; abdomen 1.68 long, 1.05 wide. As in male, except as noted. Epigastric area (Fig. 2G and Fig. 3D): externally without special features. Endogyne (Fig. 3E): receptacle broadly oval, globular appendix (gap) narrow, with anterior paddle-like sclerite (psc) and nail-like process (na), lateral sclerites functioning as muscle attachments.

#### Diagnosis

**Figure 4.**
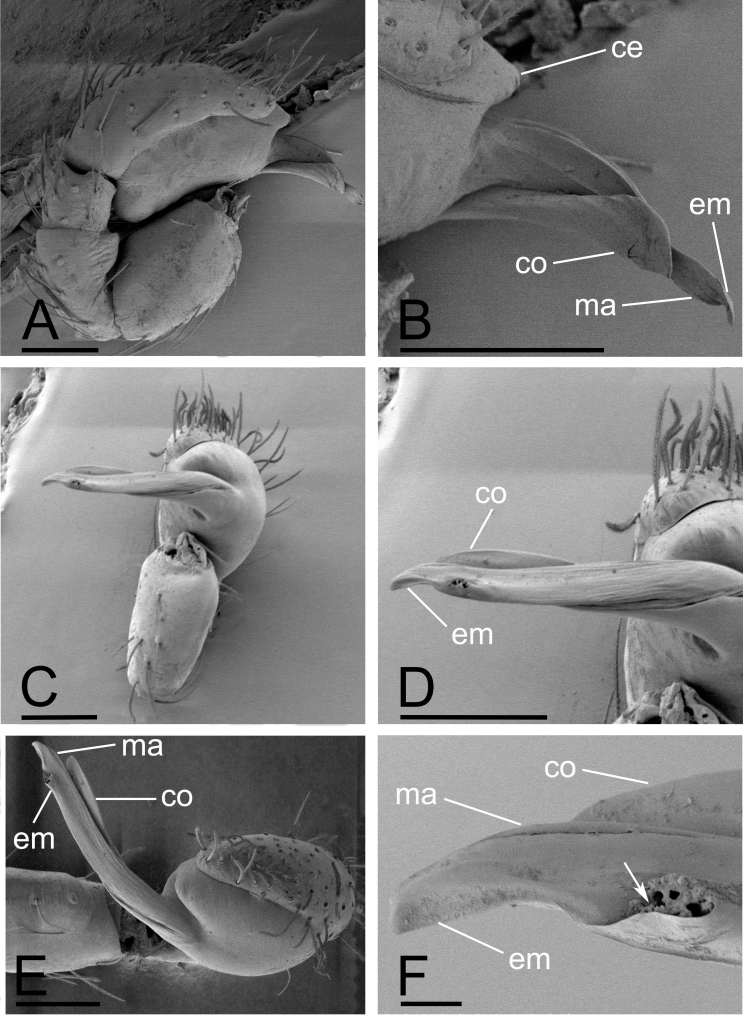
*Gamasomorpha changning* sp. nov, male left palp, SEM. **A, C, E** prolateral, ventral and retrodistal views; **B, D, F** distal part of bulb, prolateral, ventral and retrodistal views, arrow shows the opening. Abbreviations: ce = conical extension; co = conductor; em = embolus; ma = mesal embolic accessory appendage. Scale bars: 0.1 mm (A–E); 0.01 mm (F).

The new species is similar to *Gamasomorpha barbifera* Tong & Li, 2007 in the shape of the palpal bulb, but can be distinguished by the absence of a dense cluster of long setae on the male chelicerae (vs. presence of long setae; Fig. 1E, F and Tong and Li (2007): figs. 28 and 31), smooth surface of the mesal embolic accessory appendage (vs. a scale-like surface; Fig. 4A, B and Tong and Li (2007): figs. 34 and 35), a granulated carapace (vs. smooth; Fig. 1B, Fig. 2B and Tong and Li (2007): fig. 31) and large booklung covers (vs. very small; Fig. 1G, H, Fig. 2G, H and Tong and Li (2007): figs. 28 and 32).

#### Etymology

The specific name is a noun in apposition, derived from Changning, where the holotype was collected.

#### Distribution

**Figure 5.**
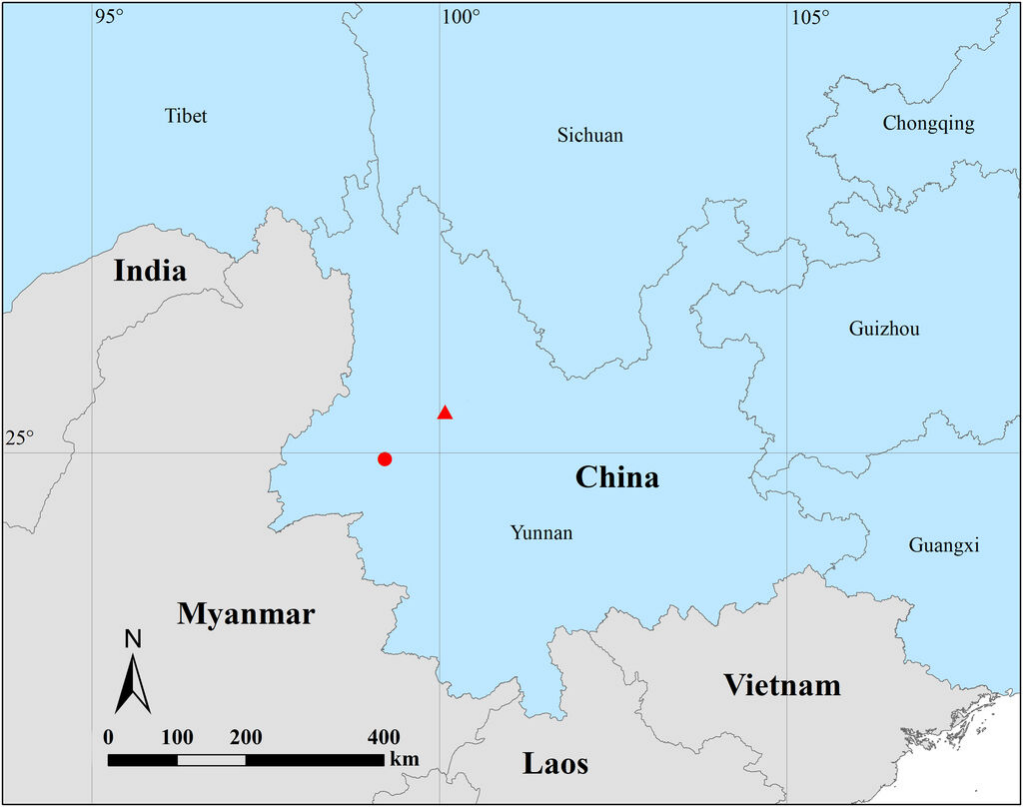
Distribution records of *Gamasomorpha changning* sp. nov. from Yunnan, China. Circle, Changning; triangle, Cangshan Mountain.

Known only from the two listed localities (Fig. 5).

## Supplementary Material

XML Treatment for Gamasomorpha changning
